# Structures of *Trichomonas vaginalis* macrophage migratory inhibitory factor

**DOI:** 10.1107/S2053230X24011105

**Published:** 2024-11-27

**Authors:** Aruesha Srivastava, Aryana Nair, Omolara C. O. Dawson, Raymond Gao, Lijun Liu, Justin K. Craig, Kevin P. Battaile, Elizabeth K. Harmon, Lynn K. Barrett, Wesley C. Van Voorhis, Sandhya Subramanian, Peter J. Myler, Scott Lovell, Oluwatoyin A. Asojo, Rabih Darwiche

**Affiliations:** a California Institute of Technology 1200 East California Boulevard Pasadena CA91125 USA; b Reedy High School, 3003 Stonebrook Parkway, Frisco, Texas, USA; c Grafton High School, 403 Grafton Drive, Yorktown, Virginia, USA; d Protein Structure and X-ray Crystallography Laboratory, 2034 Becker Drive, Lawrence, KS66047, USA; e Seattle Structural Genomics Center for Infectious Diseases, Seattle, Washington, USA; f Center for Emerging and Re-emerging Infectious Diseases (CERID), Division of Allergy and Infectious Diseases, Department of Medicine University of Washington Seattle Washington USA; g NYX, New York Structural Biology Center Upton New York USA; h Center for Global Infectious Disease Research, Seattle, Washington, USA; i Dartmouth Cancer Center, One Medical Center Drive, Lebanon, NH03756, USA; j Department of Biology University of Fribourg Chemin du Musée 10 1700Fribourg Switzerland; k Department of Biological Chemistry and Molecular Pharmacology Harvard Medical School Boston MA02115 USA; l Suliman S. Olayan School of Business, American University of Beirut, PO Box 11-0236, Riad El-Solh, Beirut, Lebanon; University of York, United Kingdom

**Keywords:** trichomoniasis, macrophage migration inhibitory factor, *Trichomonas vaginalis*, Seattle Structural Genomics Center for Infectious Disease, sexually transmitted diseases, cancer

## Abstract

The unicellular parasitic protozoan *Trichomonas vaginalis* causes trichomoniasis, the most prevalent nonviral sexually transmitted disease globally. *T. vaginalis* evades host immune responses by producing homologs of host proteins, including cytokines such as macrophage migration inhibitory factor. *T. vaginalis* macrophage migration inhibitory factor (*Tv*MIF) helps to facilitate the survival of *T. vaginalis* during nutritional stress conditions, increases prostate cell proliferation and invasiveness, and induces inflammation-related cellular pathways, thus mimicking the ability of human MIF to increase inflammation and cell proliferation. The production, crystallization and three structures of N-terminally hexahistidine-tagged *Tv*MIF reveal a prototypical MIF trimer with a topology similar to that of human homologs (hMIF-1 and hMIF-2). The N-terminal tag obscures the expected pyruvate-binding site. The similarity of *Tv*MIF to its human homologs can be exploited for structure-based drug discovery.

## Abstract 2

The production, crystallization and three crystal structures of macrophage migratory inhibitory factor from *T. vaginalis* are reported.

## 1. Introduction

*Trichomonas vaginalis* is a unicellular parasitic protozoan that is responsible for trichomoniasis, the most prevalent nonviral sexually transmitted disease globally (Edwards *et al.*, 2016 ▸). There are ∼156 million new cases of trichomoniasis worldwide each year (Molgora *et al.*, 2023 ▸). According to the Centers for Disease Control and Prevention, ∼2.6 million people in the USA have trichomoniasis, and population studies show that the highest incidence is among incarcerated women (https://www.cdc.gov/std/treatment-guidelines/trichomoniasis.htm). Humans are the only *T. vaginalis* hosts, and trichomoniasis increases susceptibility to HIV, infertility, preterm birth, HPV, and prostate cancer (Tsang *et al.*, 2019 ▸; Van Gerwen & Muzny, 2019 ▸; Zhang *et al.*, 2022 ▸). Despite its clinical significance, the molecular mechanisms underlying the development, immune evasion and host–parasite interactions of *T. vaginalis* remain poorly understood. *T. vaginalis* is a priority infectious disease for structural studies by the Seattle Structural Genomics Center for Infectious Disease (SSGCID). *T. vaginalis* evades host immune responses by producing homologs of host proteins, including cytokines such as macrophage migration inhibitory factor (MIF; Twu *et al.*, 2014 ▸). Protozoan parasite MIF homologs mimic their human MIF counterparts (hMIF-1, NCBI Accession No. CAG30406.1; hMIF-2, NCBI Accession No. CAG30317.1), facilitating the modulation of host immune responses and suppressing apoptosis-induced cell death (Twu *et al.*, 2014 ▸; Ghosh *et al.*, 2019 ▸). The human MIFs (hMIF-1 and hMIF-2) share ∼35% sequence identity, while *T. vaginalis* macrophage migration inhibitory factor (*Tv*MIF) shares ∼31% sequence identity with hMIF-1 and hMIF2. It has previously been demonstrated that *Tv*MIF elicits antibodies in infected individuals, increases prostate cell proliferation, and invasiveness, and induces inflammation-related cellular pathways, thus mimicking the ability of human MIF to increase inflammation and cell proliferation (Twu *et al.*, 2014 ▸). Furthermore, *Tv*MIF has been shown to enhance the survival of *Trichomonas* during nutritional stress conditions (Chen *et al.*, 2018 ▸). Thus, *Tv*MIF facilitates parasite survival during infection and binds to the human CD74 MIF receptor, triggering epithelial cell inflammation and proliferation pathways linked to the progression and pathogenesis of prostate cancer (Twu *et al.*, 2014 ▸; Tsang *et al.*, 2019 ▸). Here, we present the purification, crystallization and structural and functional analysis of *Tv*MIF as a first step towards uncovering features that mediate its functions.

## 2. Materials and methods

### 2.1. Macromolecule production

*Tv*MIF was cloned, expressed, and purified as described previously (Bryan *et al.*, 2011 ▸; Choi *et al.*, 2011 ▸; Serbzhinskiy *et al.*, 2015 ▸). The full-length gene for a putative macrophage migration inhibitory factor from *T. vaginalis* ATCC PRA-98/G3 (UniProt A2DXT4) encoding amino acids 1–115 was PCR-amplified from gDNA using the primers shown in Table 1 ▸. The gene was cloned into the pET-28a expression vector with an N-terminal histidine tag. The plasmid DNA was transformed into chemically competent *Escherichia coli* BL21(DE3) Rosetta cells. After testing for expression, 2 l of culture was grown using auto-induction medium (Studier, 2005 ▸) in a LEX Bioreactor (Epiphyte Three) as described previously (Serbzhinskiy *et al.*, 2015 ▸). The expression clone is available for request online at https://www.ssgcid.org/available-materials/expression-clones/.

N-terminally hexahistidine-tagged*Tv*MIF (His-*Tv*MIF) was purified using a previously described two-step protocol consisting of an immobilized metal (Ni^2+^) affinity chromatography (IMAC) step followed by size-exclusion chromatography (SEC) on an ÄKTApurifier 10 (GE Healthcare) using automated IMAC and SEC programs (Serbzhinskiy *et al.*, 2015 ▸). Briefly, thawed bacterial pellets (25 g) were lysed by sonication in 200 ml lysis buffer [25 m*M* HEPES pH 7.0, 500 m*M* NaCl, 5%(*v*/*v*) glycerol, 0.5%(*w*/*v*) CHAPS, 30 m*M* imidazole, 10 m*M* MgCl_2_, 400 µg ml^−1^ lysozyme, 3 U ml^−1^ Benzonase]. After sonication, the crude lysate was treated with 20 ml (25 U ml^−1^) of Benzonase and incubated with mixing for 45 min at room temperature. The lysate was clarified by centrifugation at 10 000 rev min^−1^ for 1 h using a Sorvall centrifuge (Thermo Scientific). The clarified supernatant was then passed over an Ni–NTA HisTrap FF 5 ml column (GE Healthcare) which had been pre-equilibrated with wash buffer [25 m*M* HEPES pH 7.0, 500 m*M* NaCl, 5%(*v*/*v*) glycerol, 30 m*M* imidazole]. The column was washed with 20 column volumes (CV) of wash buffer and eluted with elution buffer (20 m*M* HEPES pH 7.0, 500 m*M* NaCl, 5%(*v*/*v*) glycerol, 500 m*M* imidazole) over a 7 CV linear gradient.

The peak fractions were pooled and concentrated to 5 ml for SEC. The 5 ml protein sample was loaded onto a Superdex 75 26/60 column (GE Biosciences) attached to an ÄKTAprime plus FPLC system (GE Biosciences) that had been equilibrated with SEC buffer (20 m*M* HEPES pH 7.0, 300 m*M* NaCl, 5% glycerol, 1 m*M* TCEP). *Tv*MIF eluted from SEC as a single, symmetrical, monodisperse peak accounting for >90% of the protein product of molecular mass ∼19 kDa, suggesting purification as a monomer (expected monomer molecular mass of 15 kDa). The peak fractions were collected and assessed for purity by SDS–PAGE, which also suggested monomeric protein. The peak fractions were pooled and concentrated to ∼20 mg ml^−1^ using an Amicon purification system (Millipore). 110 µl aliquots of His-*Tv*MIF were flash-frozen in liquid nitrogen and stored at −80°C until use. His-*Tv*MIF protein is available for request online at https://www.ssgcid.org/available-materials/ssgcid-proteins/.

### 2.2. Crystallization

Three crystal forms of His-*Tv*MIF are reported, and all crystals were grown in UVXPO MRC (Molecular Dimensions) sitting-drop vapor-diffusion plates using the Berkeley (Pereira *et al.*, 2017 ▸; Rigaku Reagents), Index (Hampton Research) and Morpheus (Gorrec, 2009 ▸; Molecular Dimensions) crystallization screens as listed in Table 2 ▸.

### 2.3. Data collection and processing

All data sets were collected at 100 K on a Dectris EIGER2 XE 9M detector on beamline 19-ID at NSLS-II, Brookhaven National Laboratory (Table 3 ▸). Intensities were integrated using *XDS* (Kabsch, 1988 ▸, 2010 ▸) via *autoPROC* (Vonrhein *et al.*, 2011 ▸), and the Laue class analysis and data scaling were performed with *AIMLESS* (Evans, 2011 ▸). Raw X-ray diffraction images have been stored with the Integrated Resource for Reproducibility in Macromolecular Crystallo­graphy at https://www.proteindiffraction.org.

### 2.4. Structure solution and refinement

The three structures were determined by molecular replacement with *Phaser* (McCoy *et al.*, 2007 ▸) from the *CCP*4 suite of programs (Collaborative Computational Project, Number 4, 1994 ▸; Krissinel *et al.*, 2004 ▸; Winn *et al.*, 2011 ▸; Agirre *et al.*, 2023 ▸) using PDB entry 1mif (Sun *et al.*, 1996 ▸) as the search model. The structure was refined using *Phenix* (Liebschner *et al.*, 2019 ▸). Structure quality was checked with *MolProbity* (Williams *et al.*, 2018 ▸). Data-reduction and refinement statistics are shown in Table 4 ▸. Coordinates and structure factors have been deposited with the Worldwide PDB (wwPDB) as entries 8uz4 (*P*4_1_2_1_2 form), 8ur4 (*I*4_1_22 form) and 8ur2 (*I*4_1_ form). The accuracy of the ligands and waters was also checked with the *CheckMyBlob* server (Kowiel *et al.*, 2019 ▸; https://checkmyblob.bioreproducibility.org/server/).

## 3. Results and discussion

**Figure 1:**
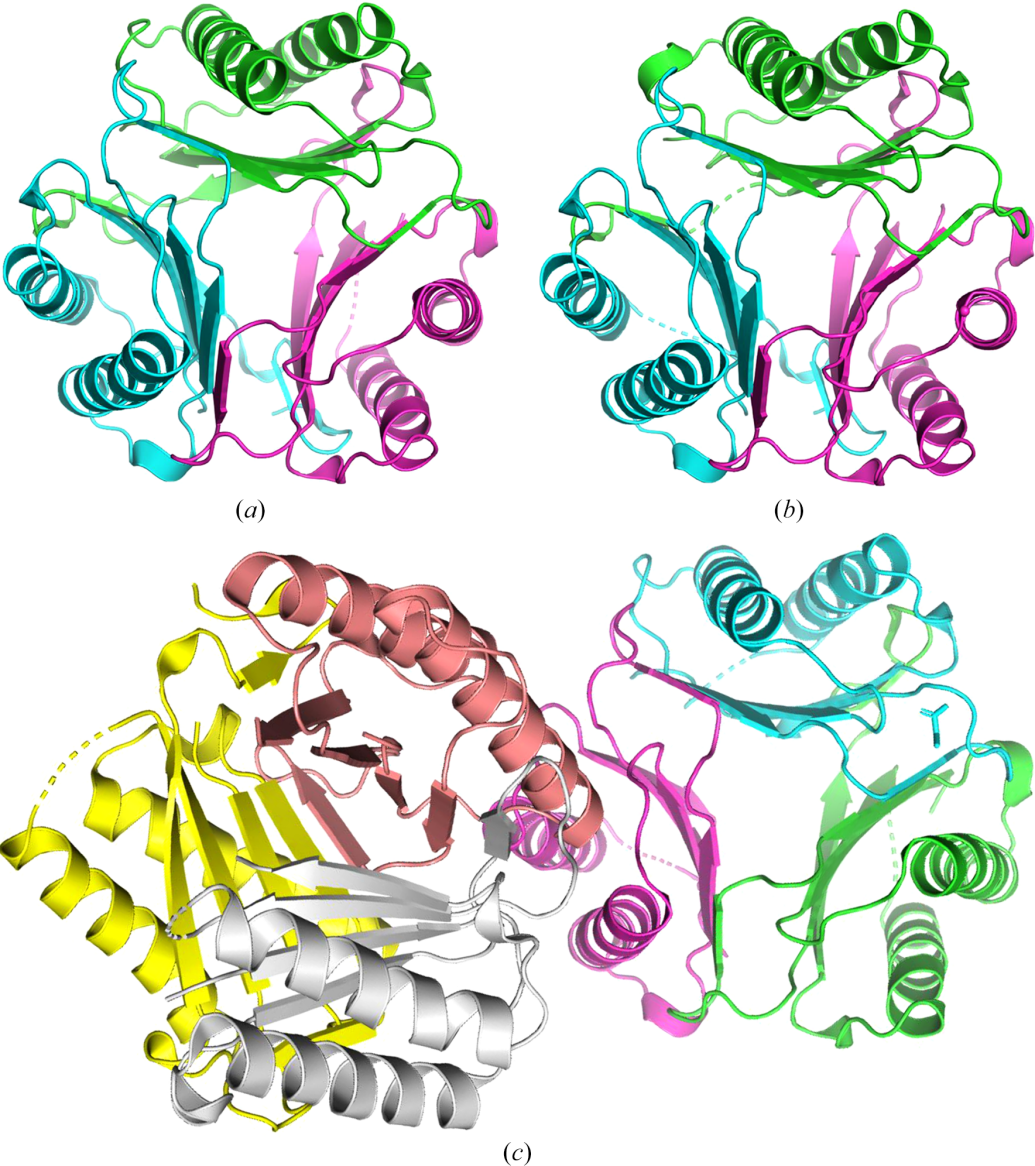
Quaternary structure of His-*Tv*MIF. All three structures reveal prototypical MIF trimers. (*a*) The apo structure (PDB entry 8uz4) and (*b*) an attempt at co-crystallization with sodium 4-hydroxyphenylpyruvate (PDB entry 8ur4) are prototypical MIF trimers. (*c*) The co-crystal with pyruvate (PDB entry 8ur2) is a dimer of two prototypical MIF trimers.

The crystallized protein, His-*Tv*MIF, included 21 additional amino-acid residues at the N-terminus corresponding to the purification tag and cleavage site (Table 1 ▸). Structures of His-*Tv*MIF were determined in three different space groups (Table 4 ▸). The first is a *P*-centered tetragonal structure (PDB entry 8uz4) with no additional density for ligands, as was expected. It has three monomers in the asymmetric unit corresponding to the prototypical MIF trimer (Fig. 1 ▸*a*). Each monomer was refined with 115 amino acids. Attempts at co-crystallization with sodium 4-hydroxyphenylpyruvate resulted in an apo structure, determined in the tetragonal space group *I*4_1_22 (PDB entry 8ur4), that contains three monomers per asymmetric unit (Fig. 1 ▸*b*).

**Figure 2:**
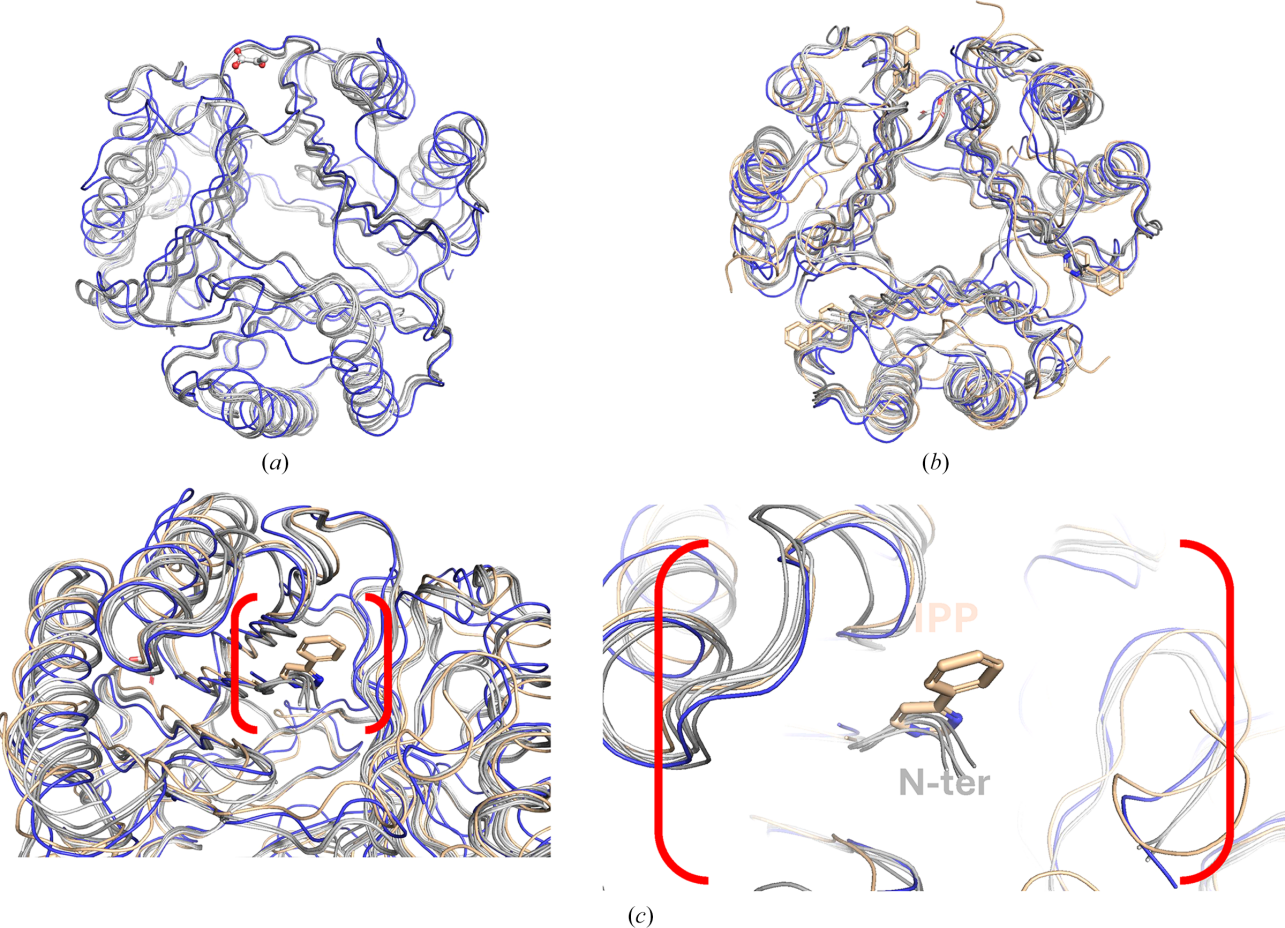
Comparison with human MIF homologs. (*a*) His-*Tv*MIF trimers (shown in gray) superpose well with each other and with hMIF-1 (PDB entry 1mif, shown in blue). (*b*) They also superpose well with hMIF-2 (PDB entry 3ker). All three structures reveal prototypical MIF trimers. (*c*) The additional residues in His-*Tv*MIF occupy the location of the tautomerase inhibitor IPP (shown as golden sticks) in hMIF-2; further details of the N-terminus are shown in the enlarged red parentheses.

The third structure was co-crystallized with pyruvate (PDB entry 8ur2) and determined in the tetragonal space group *I*4_1_. This structure is a hexamer or dimer of the prototypical MIF trimer (Fig. 1 ▸*c*). The six monomers include two with 115 amino acids, two with 100 amino acids, one with 102 amino acids and one with 99 amino acids. Analysis of all three structures with the *Protein Interfaces, Surfaces and Assembly* service (*PISA*) at the European Bioinformatics Institute (https://www.ebi.ac.uk/pdbe/prot_int/pistart.html) suggests that *Tv*MIF forms a stable prototypical MIF trimer (Krissinel, 2015 ▸). All three structures contain prototypical MIF trimers (Fig. 1 ▸*d*) that superpose with human hMIF-1 (PDB entry 1mif; Fig. 2 ▸*a*) and hMIF-2 (PDB entry 3ker; Fig. 2 ▸*b*). The r.m.s.d. for superposing C^α^ atoms of *Tv*MIF trimers with the hMIF-1 trimer is ∼1.1 Å, while that with hMIF-2 is ∼1.2 Å. *PDBeFold* (https://www.ebi.ac.uk/msd-srv/ssm/) analysis (Krissinel & Henrick, 2004 ▸) using a default threshold of 70% was used to identify the closest structural neighbors of *Tv*MIF as hMIF-1 and MIFs from other infectious protozoa, notably *Entamoeba histolytica* (PDB entry 6cuq; Seattle Structural Genomics Center for Infectious Disease, unpublished work) and *Toxoplasma gondii* (PDB entry 4dh4; Sommerville *et al.*, 2013 ▸).

The hMIF-2 structure has an inhibitor, 4-IPP, bound in the tautomerase inhibitory site in the amino-terminus. The N-terminal residues of His-*Tv*MIF obscure this inhibitor-binding site, which is otherwise accessible in both hMIF-1 and hMIF-2 (Fig. 2 ▸*c*). The obstruction by the N-terminal extension explains why 4-hydroxyphenylpyruvate does not co-crystallize with His-*Tv*MIF since its expected binding site is blocked. The obstruction also explains why pyruvate does not bind in the expected tautomerase site. A similar obstructed N-terminus was observed in the recently reported structure of *Onchocerca volvulus* MIF (Kimble *et al.*, 2024 ▸).

**Figure 3:**
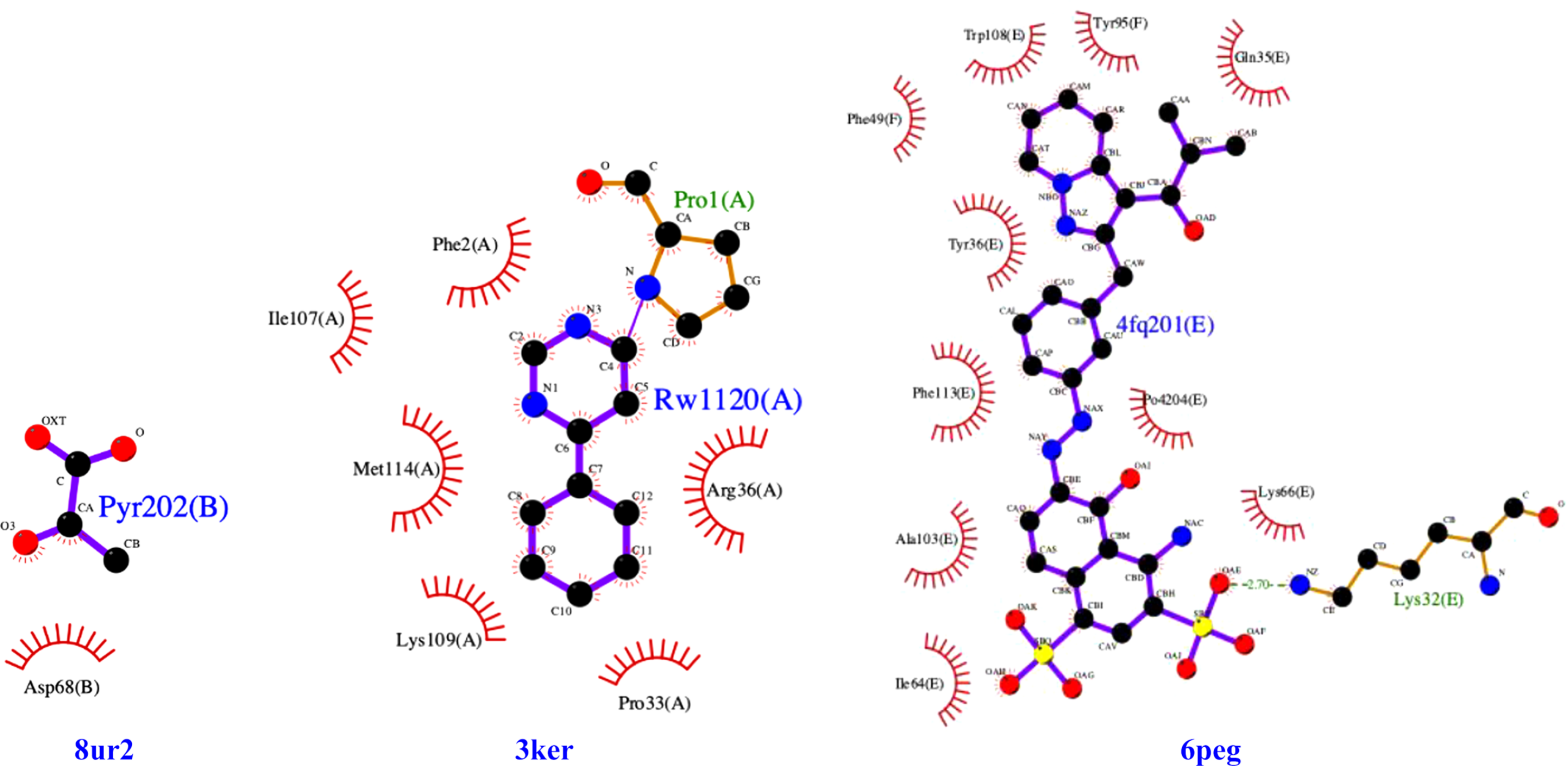
Ligand-interaction plots generated with *LigPlus* show different amino acids involved in the binding of pyruvate (Pyr202) by His-*Tv*MIF (PDB entry 8ur2), of IPP (RW1120) by hMIF-2 (PDB entry 3ker) and of an allosteric inhibitor (4fq201) by hMIF-1 (PDB entry 6peg).

The *F*_o_ − *F*_c_ omit electron-density maps of PDB entry 8ur2 can be modeled with pyruvate (Supplementary Fig. S1*a*). The location of the density is different from previously identified MIF pyruvate-binding sites, which are always at the His-tag-obscured N-terminus. We checked whether another molecule from the protein purification or crystallization solution was bound instead of pyruvate. However, pyruvate matches better than MPD or glycerol (Supplementary Fig. S1*b*). *LigPlus* analysis reveals that only one amino acid, Asp68, interacts with the pyruvate (Fig. 3 ▸). Furthermore, Asp68 is not conserved among MIFs. The location differs from the previously identified hMIF-1 allosteric inhibitor site (PDB entry 6peg; Cirillo *et al.*, 2020 ▸; Fig. 3 ▸*b*) and that of 4-IPP in hMIF-2 (PDB entry 3ker; Rajasekaran *et al.*, 2014 ▸; Fig. 3 ▸*c*). Further analysis is required to determine whether this newly identified pyruvate-binding site is biologically relevant or merely a crystallization artifact, as suggested by *CheckMyBlob*.

It has previously been demonstrated that recombinant *Tv*MIF has tautomerase activity and mimics the ability of human MIF to increase inflammation and cell proliferation (Twu *et al.*, 2014 ▸). These studies were performed with carboxyl-terminally hexahistidine-tagged *Tv*MIF, leaving the N-terminus unobstructed (Twu *et al.*, 2014 ▸). Future studies will include removal of the N-terminal tag and the generation of co-crystal structures of untagged *Tv*MIF-1 with known MIF inhibitors.

## 4. Conclusion

The production, crystallization and three structures of N-terminally hexahistidine-tagged *Tv*MIF (His-*Tv*MIF) reveal a prototypical MIF trimer with a topology similar to that of the human homologs (hMIF-1 and hMIF-2). The N-terminal tag obscures the expected pyruvate-binding site. The similarity of *Tv*MIF to its human homologs and to other MIFs (Supplementary Fig. S2) can be exploited for structure-based drug discovery.

## Supplementary Material

PDB reference: macrophage migration inhibitory factor, space group *I*4_1_, 8ur2

PDB reference: space group *I*4_1_22, 8ur4

PDB reference: space group *P*4_1_2_1_2, 8uz4

Supplementary Figures. DOI: 10.1107/S2053230X24011105/ir5039sup1.pdf

## Figures and Tables

**Table 1:** Macromolecule-production information

Source organism	*Trichomonas vaginalis* ATCC PRA-98/G3
DNA source	CollegeCodon optimized and synthetically generated plasmid from Twist Bioscience
Expression vector	pET-28a, AVA N-terminal tag
Expression host	*Escherichia coli* BL21(DE3) Rosetta
Complete amino-acid sequence of the construct produced†	**MAHHHHHHMGTLEAQTQGPGS**MPALVIKTNAKFTEEEKSKATEELGNIVSKVLGKPISYVMVTLEDGVAVRFGGSDEKAAFMSLMSIGGLNRAVNKRASAALTKWFTDHGFQGDRIYIVFNPKSAEDWGFNGDTFA

† The additional N-terminal amino acid residues are in bold.

**Table 2:** Crystallization

Crystal form	PDB entry 8uz4, apo, *P*4_1_2_1_2	PDB entry 8ur4, *I*4_1_22	PDB entry 8ur2, *I*4_1_
Temperature (K)	291	291	291
Protein concentration (mg ml^−1^)	35.4	35.4	35.4
Buffer composition of protein solution	20 m*M* HEPES pH 7.0, 300 m*M* NaCl, 5% glycerol, 1 m*M* TCEP	20 m*M* HEPES pH 7.0, 300 m*M* NaCl, 5% glycerol, 1 m*M* TCEP, 5 m*M* sodium 4-hydroxyphenylpyruvate	20 m*M* HEPES pH 7.0, 300 m*M* NaCl, 5% glycerol, 1 m*M* TCEP, 5 m*M* sodium pyruvate
Composition of reservoir solution	Berkeley D5: 100 m*M* HEPES free acid/sodium hydroxide pH 7.5, 200 m*M* ammonium acetate, 25%(*w*/*v*) PEG 3350	Index A4: 0.1 *M* bis-Tris pH 6.5, 2.0 *M* ammonium sulfate	Morpheus B12: 12.5%(*v*/*v*) MPD, 12.5%(*v*/*v*) PEG 1000, 12.5%(*w*/*v*) PEG 3350, 100 m*M* Tris–Bicine pH 8.5, 30 m*M* NaF, 30 m*M* NaBr, 30 m*M* NaI
Volume and ratio of drop	0.2 µl, 1:1	0.2 µl, 1:1	0.2 µl, 1:1
Volume of reservoir (µl)	40	40	40
Composition of cryoprotectant solution	80 m*M* HEPES free acid/sodium hydroxide pH 7.5, 160 m*M* ammonium acetate, 20%(*w*/*v*) PEG 3350, 20%(*v*/*v*) PEG 200	2.5 *M* lithium sulfate, 0.1 *M* bis-Tris pH 6.5, 2.0 *M* ammonium sulfate, 20%(*w*/*v*) PEG 3350, 20%(*v*/*v*) PEG 200	Directly from crystallization buffer

**Table 3:** Data collection and processing Values in parentheses are for the outer shell.

Data set	PDB entry 8uz4, apo, *P*4_1_2_1_2	PDB entry 8ur4, *I*4_1_22	PDB entry 8ur2, *I*4_1_
Temperature (K)	100	100	100
Space group	*P*4_1_2_1_2	*I*4_1_22	*I*4_1_
*a*, *b*, *c* (Å)	80.72, 80.72, 121.15	109.87, 109.87, 125.88	118.39, 118.39, 106.66
α, β, γ (°)	90, 90, 90	90, 90, 90	90, 90, 90
Resolution range (Å)	80.71–2.40 (2.46–2.40)	82.78–2.55 (2.62–2.55)	83.71–1.90 (1.95–1.90)
Total No. of reflections	379848 (29417)	211801 (15124)	787413 (58149)
Completeness (%)	100 (100)	100 (100)	100 (100)
Multiplicity	23.2 (24.6)	16.4 (15.8)	13.6 (13.6)
〈*I*/σ(*I*)〉	21.2 (1.6)	14.8 (1.6)	16.6 (1.8)
*R*_r.i.m._	0.093 (2.75)	0.113 (2.02)	0.078 (1.68)
*R*_p.i.m._	0.020 (0.55)	0.033 (0.51)	0.021 (0.45)

**Table 4:** Structure refinement Values in parentheses are for the outer shell.

Structure	PDB entry 8uz4, apo, *P*4_1_2_1_2	PDB entry 8ur4, *I*4_1_22	PDB entry 8ur2, *I*4_1_
Resolution range (Å)	67.17–2.40 (2.55–2.40)	82.78–2.55 (2.75–2.55)	83.71–1.90 (1.93–1.90)
Completeness (%)	100 (100)	100 (100)	99.8 (99.8)
No. of reflections			
Working set	16289 (2517)	12901 (2400)	57681 (2599)
Test set	816 (131)	646 (126)	2990 (145)
Final *R*_cryst_	0.245 (0.397)	0.253 (0.339)	0.192 (0.341)
Final *R*_free_	0.264 (0.431)	0.280 (0.416)	0.230 (0.383)
No. of non-H atoms			
Protein	2588	2576	4713
Ion	0	5	1
Ligand	0	0	6
Water	3	0	167
Total	2591	2581	4887
R.m.s. deviations			
Bond lengths (Å)	0.008	0.003	0.011
Angles (°)	1.124	0.501	0.909
Average *B* factors (Å^2^)			
Protein	96.3	90.9	54.4
Ion	0	88.5	124.4
Ligand	0	0	60.0
Water	61.9	0	47.5
Ramachandran plot (%)			
Favored regions	99	98	99
Additionally allowed	1	2	1
Outliers	0	0	0
