# Supplementary material for: Structures of Trichomonas vaginalis macrophage migratory inhibitory factor
Source: Acta Crystallogr F Struct Biol Commun. 2024 Nov 27;80(Pt 12):341–7. doi: 10.1107/S2053230X24011105 (PMC11614108; doi:10.1107/S2053230X24011105)
Supplement: Supplementary file 1 [file f-80-00341-sup1.pdf]

STRUCTURAL BIOLOGY COMMUNICATIONS

Volume 80 (2024)

Supporting information for article:

Structures of *Trichomonas vaginalis* macrophage migratory inhibitory factor

Aruesha Srivastava, Aryana Nair, Omolara C. O. Dawson, Raymond Gao, Lijun Liu, Justin K. Craig, Kevin P. Battaile, Elizabeth K. Harmon, Lynn K. Barrett, Wesley C. Van Voorhis, Sandhya Subramanian, Peter J. Myler, Scott Lovell, Oluwatoyin A. Asojo and Rabih Darwiche

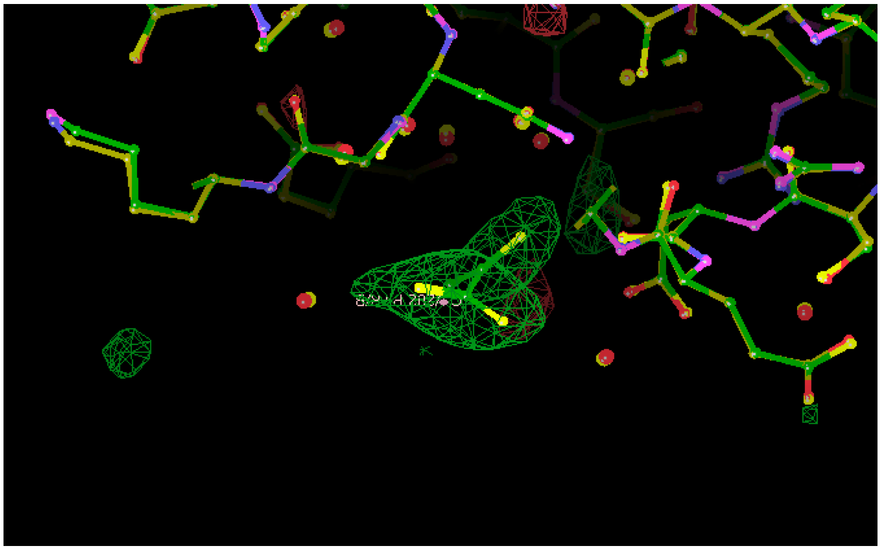

(a)

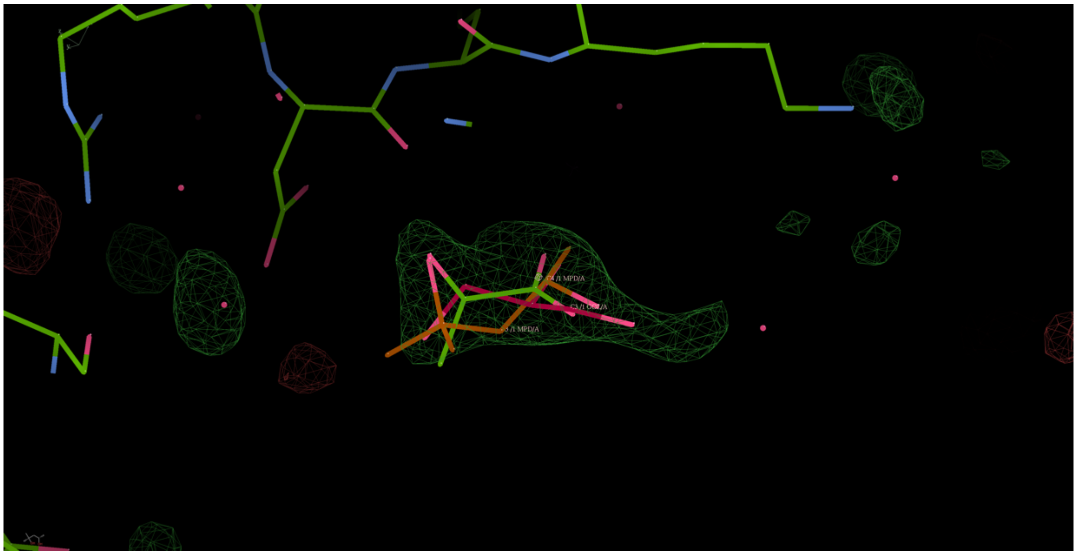

(b)

Figure S.1 a) Pyruvate fits into the difference (Fo-Fc) omit electron density maps (green mesh) contoured at 3.0 sigma. b) A comparison of glycerol and MPD binding to the omit electron density maps reveals that pyruvate fits better.

Figure S.2 Blast alignment against the PDB of TvMIF

NCBI Multiple Sequence Alignment Viewer, Version 1.25.2

Sequence ID	Start	Alignment	End	Organism
Query_8936939 (+)	1	MPALVIKTNAKFTEEEKSKATEELGNIVSKVLGKPISYVMVTLEDGVAVRFGGSDEKAAFMSLMSIGGLNRAVNKRASAALTKWFTDHGFQGDRIYIVFNPKSAEDWGFNGDTFA	115	
8UR2_A (+)	22	....	136	Trichomonas vaginalis G3
3T5S_A (+)	22	..CAIVT...D..KDQADAFCLDM.QVLA.ET...V..C.AGVRKA-DMS..T.TDLCC.VDFYC..VISQ.K.PSI...I.GCL.Q.KVKPE.V..S..EAKGHN.......S..	135	Giardia lamblia ATCC 5...
3GAC_A (+)	1	.CCELI..ISIPDDKAQN.LS.IEDAI.N.....VA.I.SNYDYQKNL..S..N.GYC.VR.T.....I..SN.SSLADKI..ILSN..VKPR.V..E.RDC..QNFA.S.SL.	114	Plasmodium yoelii yoelii
9B0M_A (+)	22	..CCQVS..INASDDDAK..LSQIE.AI.Q......LG.I.SN.DYQKHM......HDGFC.VRVT.L..I.KSN.SSLADKI..ILASTNVKSE.VF.E.KDC..QNFA....SL.	136	Plasmodium vivax Sal-1
2WKB_A (+)	1	.XCELI..ISIPDDKAQNTLS.IEDAI.NI....VA.I.SNYDYQKNL..S..N.GYC.VR.T.....I..SN.SLLADKI..ILSN.SVKPR.V..E.RDC..QNFA.S.SL.	114	Plasmodium berghei A...
4P7M_A (+)	1	.CCEVI..VNLPDDNVQSTLSQIE.AI.D.M...LG.I.SNYDYQKNL.....N.AYC.VRIT....I..SN.SALADQI..LLVSNNVKSR...VE.RDC..QNFA.S.SL.	114	Plasmodium falciparum
2WKF_A (+)	1	.CCEVI..VNLPDDNVQSTLSQIE.AI.D.M...LG.I.SNYDYQKNL.....N.AYC.VRIT....I..SN.SALADQI..LLVSNNVKSR...VE.RDC..QNFA.S.SL.	114	Plasmodium falciparum ...
2WKF_B (+)	1	.XCEVI..VNLPDDNVQSTLSQIE.AI.D.M...LG.I.SNYDYQKNL.....N.AYC.VRIT....I..SN.SALADQI..LLVSNNVKSR...VE.RDC..QNFA.S.SL.	114	Plasmodium falciparum ...
8AP3_A (+)	1	.C.NLS..VNLDGVDT.SILS.ASST.A.II...EN...IV.KGS.PMS...TEDP..YGE.V.......AD...KL...VSAIL.KLSVPKS.FFLK.YDTKGSFF.W..A.L	114	Arabidopsis thaliana
4DH4_A (+)	1	.KCM.FCPVAA.PAQQDALLKDAEKA.ADA.....L......GYSQTGQM.....SDPC..IRVA.....ITSST.CKIA...SAACER..VPKN....TT.TN..PSE.AMGDR..	114	Toxoplasma gondii
2XCZ_A (+)	1	..LIN.QASVPAVADAN.-LLQ..SSKLAEL.....EK...TS.QC..PMT.S.NT.PTCYVEVK...A.DGSRTQEV.ELVCGHIEQN.IPA......G.EDVP.RL..W..S..	114	Prochlorococcus marin...
1HFO_A (+)	1	.IFTLN..I.A.DVP-.DFLSSTSAL.GNI.S..G...A.HINTDQQLS.....TNP...GT......IEPSR.RDH..K.FDHL.KL.IPKN.M..H.VNLNGD.V.W..T..	113	Trichinella spiralis
8VJ2_A (+)	22	...FT.N..-IPQSNV.D.LKKASST.A.A.....E...AIHVNG.Q.MV.....TDPC.VCV.K...CVGPN..NSH.EK.F.LLA.EKIPKN.C..E.VNID.STMA...S..	135	Onchocerca volvulus
8DQ6_A (+)	1	.T.NLF..IPVDAVTC.DILKDATKA.A.II....E.....IL.NS..PIA.A.TE.P..YGE.I......GPG..GKL.ETISEILIKLSIDSS.F..K.YDSPRPFF.Y	109	Arabidopsis thaliana
6ICE_A (+)	1	..MFTVN...SVP.GLL.EL.QQ.-AQAT...AQ.IA.HVVPDQLMT.S..SDPC.LC..H...KIGG.Q.RTY.KL.CGLLA.RHISP......NYYDM..ANV.W..S...	115	Mesocricetus auratus
1FIM_A (+)	1	...FIVN...SVP.GFL.EL.QQ.-AQAT...AQ.IA.HVVPDQLXT.S.TSDPC.LC..H...KIGG.Q.RNY.KL.CGLLS.RHISP..V..NYYDAN.ANV.W..S...	114	Rattus norvegicus
1MFI_A (+)	1	.MFIVN...SVP.GFL.EL.QQ.-AQAT...AQ.IA.HVVPDQLMT.S.TNDPC.LC..H...KIGG.Q.RNY.KL.CGLLS.RHISP..V..NYYDMN.ANV.W..S...	114	Mus musculus
1MFF_A (+)	1	.MFIVN...SVP.GFL.EL.QQ.-AQAT...AQ.IA.HVVPDQLMT.S.TNDPC.LC..H...KIGG.Q.RNY.KL.CGLLS.RHISP..VF.NYYDMN.ANV.W..S...	114	Mus musculus
1UIZ_A (+)	1	..VFT.R..V-CRDSVPDTLLSD.TKQLA.AT...AE.IAIHIVPDQIMS..D.TDPC.VC..C...KIGGPQ..SYTKL.CDIL.KQNIPAN.V..NYYDLN.ANV.W..S...	115	Xenopus laevis
